# Distinct non-synonymous mutations in cytochrome* b* highly correlate with decoquinate resistance in apicomplexan parasite *Eimeria tenella*

**DOI:** 10.1186/s13071-023-05988-7

**Published:** 2023-10-17

**Authors:** Zhenkai Hao, Junmin Chen, Pei Sun, Linlin Chen, Yuanyuan Zhang, Wenxuan Chen, Dandan Hu, Feifei Bi, Zhenyan Han, Xinming Tang, Jingxia Suo, Xun Suo, Xianyong Liu

**Affiliations:** 1 National Key Laboratory of Veterinary Public Health and Safety, Key Laboratory of Animal Epidemiology and Zoonosis of Ministry of Agriculture, National Animal Protozoa Laboratory & College of Veterinary Medicine, China Agricultural University, Beijing, 100193 China; 2 Key Laboratory of Animal Genetics, Breeding and Reproduction of the Ministry of Agriculture & Beijing Key Laboratory of Animal Genetics Improvement, China Agricultural University, Beijing, China; 3 School of Animal Science and Technology, Guangxi University, Guangxi, China; 4 Key Laboratory of Animal Biosafety Risk Prevention and Control (North) of MARA, Institute of Animal Sciences, Chinese Academy of Agricultural Sciences, Beijing, China

**Keywords:** *Eimeria tenella*, Drug resistance, Decoquinate, Whole-genome sequencing, Cytochrome* b*, Non-synonymous mutation

## Abstract

**Background:**

Protozoan parasites of the genus *Eimeria* are the causative agents of chicken coccidiosis. Parasite resistance to most anticoccidial drugs is one of the major challenges to controlling this disease. There is an urgent need for a molecular marker to monitor the emergence of resistance against anticoccidial drugs, such as decoquinate.

**Methods:**

We developed decoquinate-resistant strains by successively exposing the Houghton (H) and Xinjiang (XJ) strains of *E. tenella* to incremental concentrations of this drug in chickens. Additionally, we isolated a decoquinate-resistant strain from the field. The resistance of these three strains was tested using the criteria of weight gain, relative oocyst production and reduction of lesion scores. Whole-genome sequencing was used to identify the non-synonymous mutations in coding genes that were highly associated with the decoquinate-resistant phenotype in the two laboratory-induced strains. Subsequently, we scrutinized the missense mutation in a field-resistant strain for verification. We also employed the AlphaFold and PyMOL systems to model the alterations in the binding affinity of the mutants toward the drug molecule.

**Results:**

We obtained two decoquinate-resistant (DecR) strains, DecR_H and XJ, originating from the original H and XJ strains, respectively, as well as a decoquinate-resistant *E. tenella* strain from the field (DecR_SC). These three strains displayed resistance to 120 mg/kg decoquinate administered through feed. Through whole-genome sequencing analysis, we identified the cytochrome* b* gene (*cyt b*; ETH2_MIT00100) as the sole mutated gene shared between the DecR_H and XJ strains and also detected this gene in the DecR_SC strain. Distinct non-synonymous mutations, namely Gln131Lys in DecR_H, Phe263Leu in DecR_XJ, and Phe283Leu in DecR_SC were observed in the three resistant strains. Notably, these mutations were located in the extracellular segments of* cyt b*, in close proximity to the ubiquinol oxidation site *Q*_o_. Drug molecular docking studies revealed that* cyt b* harboring these mutants exhibited varying degrees of reduced binding ability to decoquinate.

**Conclusions:**

Our findings emphasize the critical role of* cyt b* mutations in the development of decoquinate resistance in *E. tenella*. The strong correlation observed between* cyt b* mutant alleles and resistance indicates their potential as valuable molecular markers for the rapid detection of decoquinate resistance.

**Graphical abstract:**

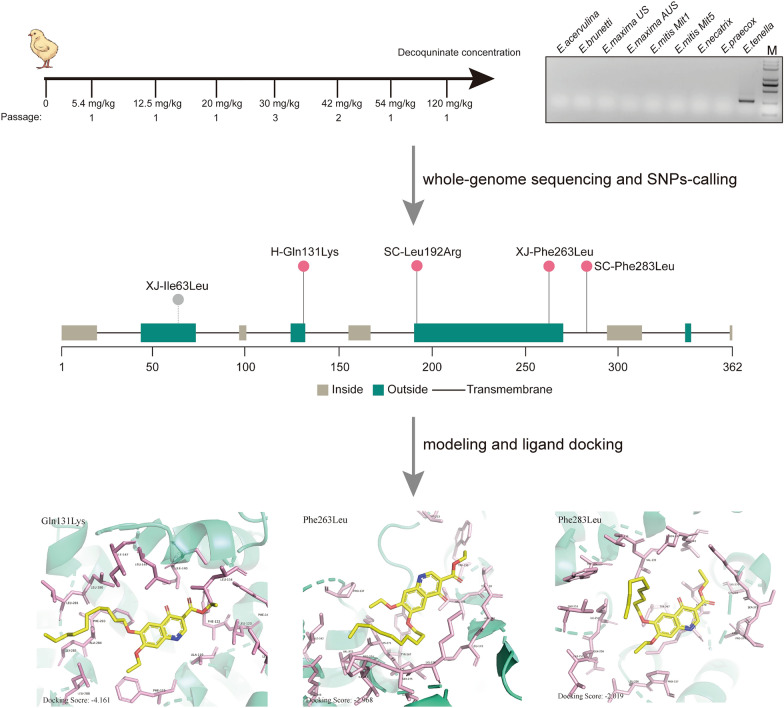

**Supplementary Information:**

The online version contains supplementary material available at 10.1186/s13071-023-05988-7.

## Background

Coccidiosis, caused by protozoan parasites of the genus *Eimeria*, is the most common disease in chickens. The control of coccidiosis relies on the use of various anticoccidial drugs, including polyether ionophore antibiotics (such as monensin, lasalocid and salinomycin) and chemicals (like halofuginone, decoquinate and diclazuril) [1]. However, prolonged and extensive use of these drugs has led to the emergence of drug resistance across nearly all anticoccidial agents, raising serious concerns regarding drug abuse [2, 3]. Currently, the primary method for identifying coccidiostat resistance is through drug susceptibility testing using chickens in laboratories, which is time-consuming and laborious [4]. Unfortunately, the genetic mechanisms underlying drug resistance are still poorly understood. Only a few studies have identified drug-resistant phenotypes by comparison of transcriptomic or proteomics data between drug-sensitive strains and drug-resistant strains [5]. As a result, no reliable tool has yet been developed or employed for the rapid detection of anticoccidial drug resistance.

Decoquinate, a quinoline derivative, is a synthetic coccidiostat widely used to prevent *Eimeria* infection in domestic chickens since its introduction in 1967 [6]. However, parasite resistance to decoquinate emerged soon after in 1970 in the UK and has since been reported worldwide [7, 8]. At the present time, decoquinate is primarily used in shuttle programs and withdrawal feeds [9]. It exhibits an anticoccidial effect on the first-generation schizonts of *E. tenella* [10] and can also promote the development of immunity when administered below its coccidiostatic threshold [11]. Previous studies have shown that decoquinate acts on the sporozoite stage of the life-cycle by disrupting electron transport in the mitochondrial cytochrome system of coccidia [12–14].

Studies investigating decoquinate-resistant *E. tenella* lines have indicated that unsporulated oocysts of these lines displayed tolerance to decoquinate inhibition while exhibiting greater sensitivity to cyanide and azide [15]. These findings suggest that drug-resistant parasites have evolved alternative pathways for electron transport in the mitochondrion. Under an experimental regimen of increasing selection pressure on *E. tenella* parasites with higher decoquinate concentrations, Chapman developed a decoquinate-resistant *E. tenella* after eight serial passages in chickens [16]. Interestingly, this resistant parasite did not lose its resistance even after 10 passages in chickens without medication [17]. Furthermore, experiments introducing an equal number of sensitive and resistant oocysts into floor pen chickens have shown that in the absence of medication, decoquinate-sensitive *E. tenella* parasites tend to dominate, suggesting that a fitness cost is associated with resistance disadvantages in resistant parasites [18]. Despite these studies providing valuable insights into the general patterns of decoquinate resistance, the genetic basis of resistance in *E. tenella* remains unexplored.

In recent years, extensive research on other apicomplexan parasites, such as *Plasmodium* spp. and *Toxoplasma gondii*, has led to the identification of several genetic markers associated with antiprotozoal agents [19]. The tools used in these studies enable us to explore the genetic mechanisms underlying *E. tenella* resistance against anticoccidial drugs like decoquinate. In the present study, we used whole-genome sequencing to identify the potential genetic markers of decoquinate resistance in *E. tenella*.

## Methods

### Animals and parasites

Ross 308 broilers aged between 1 and 6 weeks were utilized for the proliferation and development of decoquinate-resistant *E. tenella* strains. All birds were provided with a coccidia-free diet and water. *Eimeria tenella* Houghton (H) strain and Xinjiang (XJ) strain, which are sensitive to decoquinate, served as the parental strains. A third *E. tenella* strain, the SC strain, is a field-isolated strain isolated from a broiler farm in Sichuan province, China with a history of prolonged decoquinate use. The protocols for oocyst collection, sporulation and purification were as previously described [20]. Euthanasia was performed to chickens using cervical dislocation to ensure a rapid and painless loss of consciousness.

### Development of decoquinate-resistant *E. tenella* strains

**Fig. 1:**
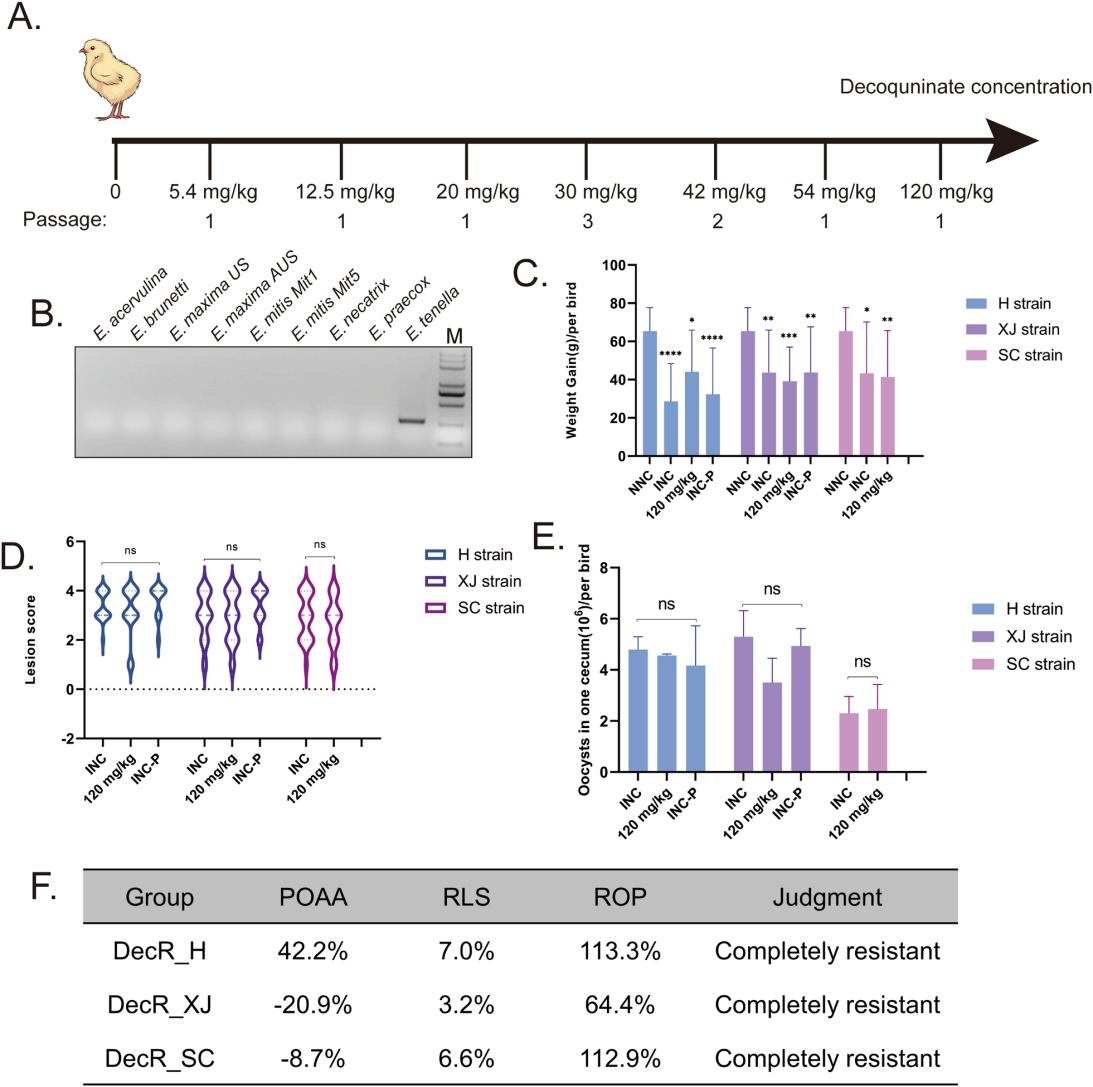
**A**,** B** Process used in the laboratory to develop decoquinate-resistant strains by gradually increasing the drug level (**A**) and identifying the *Eimeria* species isolated from the SC strain by PCR (**B**). *Eimeria maxima* US and AUS primers were designed based on internal transcribed spacer 1 (ITS-1) sequences of the US and Australian *E. maxima* isolates, respectively, and *E. mitis* Mit1 and Mit5 were designed to amplify the two different clones found in *E. mitis* isolate. M, Marker.** C**,** D**,** E** The drug resistance test results, including weight gain (**C**), lesion scores (**D**) and oocysts in one cecum per bird (**E**) were measured. Statistical analysis using ordinary one-way analysis of variance for weight gain (**C**) and t-tests for lesion scores (**D**) and oocysts in one cecum per bird (**E**). Asterisks indicate a significant difference at *P < 0.05, **P < 0.01, ***P < 0.001 and ****P < 0.0001; ns, not significant.** F** The resistance evaluation is based on three criteria: percentage of optimum anticoccidial activity (POAA; > 50% sensitivity, ≤ 50% resistance), reduction of lesion scores (RLS; > 50% sensitivity, ≤ 50% resistance) and relative oocyst production (ROP; ≥ 15% resistance, < 15% sensitivity). DecR, Decoquinate resistance; H, XJ, Houghton and Zinjiang original strains of *E. tenella*; INC, infected non-medicated controls; INC-P, parent strain non-medicated controls; NNC, non-infected, non-medicated birds; SC, decoquinate-resistant strain isolated from the field; SNP, single nucleotide polymorphism

The development of decoquinate resistance in *E. tenella* followed a previously described protocol [16]. In brief, the H and XJ strains were used to develop decoquinate resistance in groups of 10 medicated birds. Each bird received a dose of 10^5^ sporulated oocysts, with decoquinate added to the diet 24 h before parasite inoculation; the concentrations of decoquinate used during the process to develop resistance are outlined in Fig. 1A. In instances where the number of oocysts was insufficient to maintain the standard dose, the latter was prepared by successive passaging at the current drug concentration. Oocysts were collected from feces between 5 and 9 days post inoculation.

### Single-oocyst isolation of *E. tenella* in field decoquinate-resistant strains

To propagate the SC strain, chickens were administered 120 mg/kg of decoquinate in their diet and inoculated with 10^4^ oocysts at 24 h post inoculation. Fecal samples were collected on the day 6 post infection and examined for oocysts. Oocysts were collected from the caeca of birds on day 7 post infection then sporulated.

The protocol for single-oocyst isolation of *E. tenella* was adapted from a previous study [21]. In summary, sporulated oocysts were diluted in phosphate buffered saline (PBS; pH 7.4) to a concentration of approximately 4 oocysts per microliter. A glass slide fitted with transparent, thin plastic sheets was placed under a microscope, and a drop (0.25 μl) of the diluted oocysts was dropped onto the center of one plastic sheet. The entire drop was thoroughly examined at 20× magnification to confirm the presence of only one oocyst with typical *E. tenella* morphological features. If the drop was confirmed to contain only one oocyst, it was immediately covered with 3 μl of 50% glycerol (in PBS, v/v). This piece of plastic sheet containing a single oocyst was then removed from the slide with a fine forceps and given orally to a 1-day-old chicken medicated with decoquinate. A total of 10 birds were infected with a single oocyst each. Oocysts were collected from the cecal mucosa on the day 7 post infection. The oocysts were sporulated and propagated with medication in chickens.

### Genomic DNA extraction and species identification with PCR

The genomic DNA of *E. tenella* was extracted using the cetyltrimethylammonium bromide (CTAB) method, and PCR primers described in a previous publication were used for identification [22]. A nested PCR strategy was employed. The primers EF1 (aagttgcgtaaatagagccctc) and ER2 (agacatccattgctgaaag) targeted the internal transcribed spacer 1 (ITS-1) of *Eimeria* species, while the primers ETF (aatttagtccatcgcaaccct) and ETR (cgagcgctctgcatacgaca) identified *E. tenella* using the product of the first PCR as a template.

### Evaluation of decoquinate resistance of the three *E. tenella* strains

To assess the decoquinate resistance of *E. tenella* strains, 182 fourteen-day-old Ross 308 broilers were divided into three parental groups, H, XJ and SC, comprising 63, 63 and 42 chickens, respectively, as well as a control group of 14 non-infected, non-medicated birds (NNC). Each group was further divided into nine or six subgroups, each with seven birds. To serve as infected non-medicated controls (INC), chickens in groups H1-H3, XJ1-XJ3 and SC1-SC3 were inoculated but not treated with decoquinate. Chickens in groups H4-H6, XJ4-XJ6 and SC4-SC6 were medicated with 120 mg/kg of decoquinate in their feed 24 h prior to inoculation. Chickens in groups H7-H9 and XJ7-9 were inoculated with parent strains of *E. tenella* but not treated with medication, serving as parent strain non-medicated controls (INC-P). Each bird in groups H, XJ and SC was infected with 10^4^ sporulated oocysts of *E. tenella*. The body weights of the chickens were measured on the day of infection and again on day 7 post infection when they were sacrificed. The lesion scores on one side of the cecum were evaluated based on the scoring system proposed by Johnson and Reid [21], while the number of oocysts on the other side of the cecum was determined using the McMaster Chamber.

Resistance to decoquinate was evaluated using a combination of three criteria: the percentage of optimum anticoccidial activity (POAA), the reduction of lesion scores (RLS) and the relative oocyst production (ROP) [22–25]. POAA was calculated using the following formula: (growth and survival ratio [GSR] of treatment group − GSR of INCs)/(GSR of NNCs − GSR of INCs) × 100%, where GSR represents the cage weight at termination divided by the cage weight at initiation. The RLS was determined by comparing the mean lesion scores of the treatment groups to those of the INCs using the formula: (mean LS of INCs − mean LS of treatment groups)/mean LS of INCs × 100%. ROP was calculated by determining the ratio of oocyst production in the treatment groups to that of the INCs using the formula: (oocyst production of treatment groups/oocyst production of INCs) × 100%. When all three criteria above indicated resistance, the strain was judged to be ‘completely resistant.’ ‘Moderately resistant’ and ‘slightly resistant’ were defined as only two of the three criteria indicating resistance and only one of the three criteria indicating resistance being met, respectively.

### Whole-genome sequencing and data analysis

Whole-genome sequencing was performed for the following strains: H-12.5, H-30, H-54, H-120, XJ-00, XJ-12.5, XJ-30, XJ-54, XJ-120 and SC-120, using the Illumina paired-reads sequencing technology (Illumina, Inc., San Diego, CA, USA) according to the manufacturer’s instructions, which involved sample quality control, library construction, library quality assessment and library sequencing. Raw reads were filtered using Fastp [26] to obtain clean reads suitable for bioinformatics analysis. The filtered reads were then mapped to a reference genome (https://www.ncbi.nlm.nih.gov/assembly/GCA_905310635.1) with the Burrows-Wheeler Alignments Tool (BWA) [27], resulting a BAM file. Samtools [28] was used to sort the BAM files and gather statistics, including sequencing depth, genome coverage and other relevant information for each sample.

The Genome Analysis Toolkit (GATK) [29] was used to extract the single-nucleotide polymorphisms (SNPs) and generate variant call format (VCF) files for each sample. The identified SNPs were further filtered using the parameters provided in the official manual to obtain high-quality SNPs. Thereafter, Plink2 [30] was used to perform dimensionality reduction algorithms, specifically principal component analysis (PCA).

**Fig. 2:**
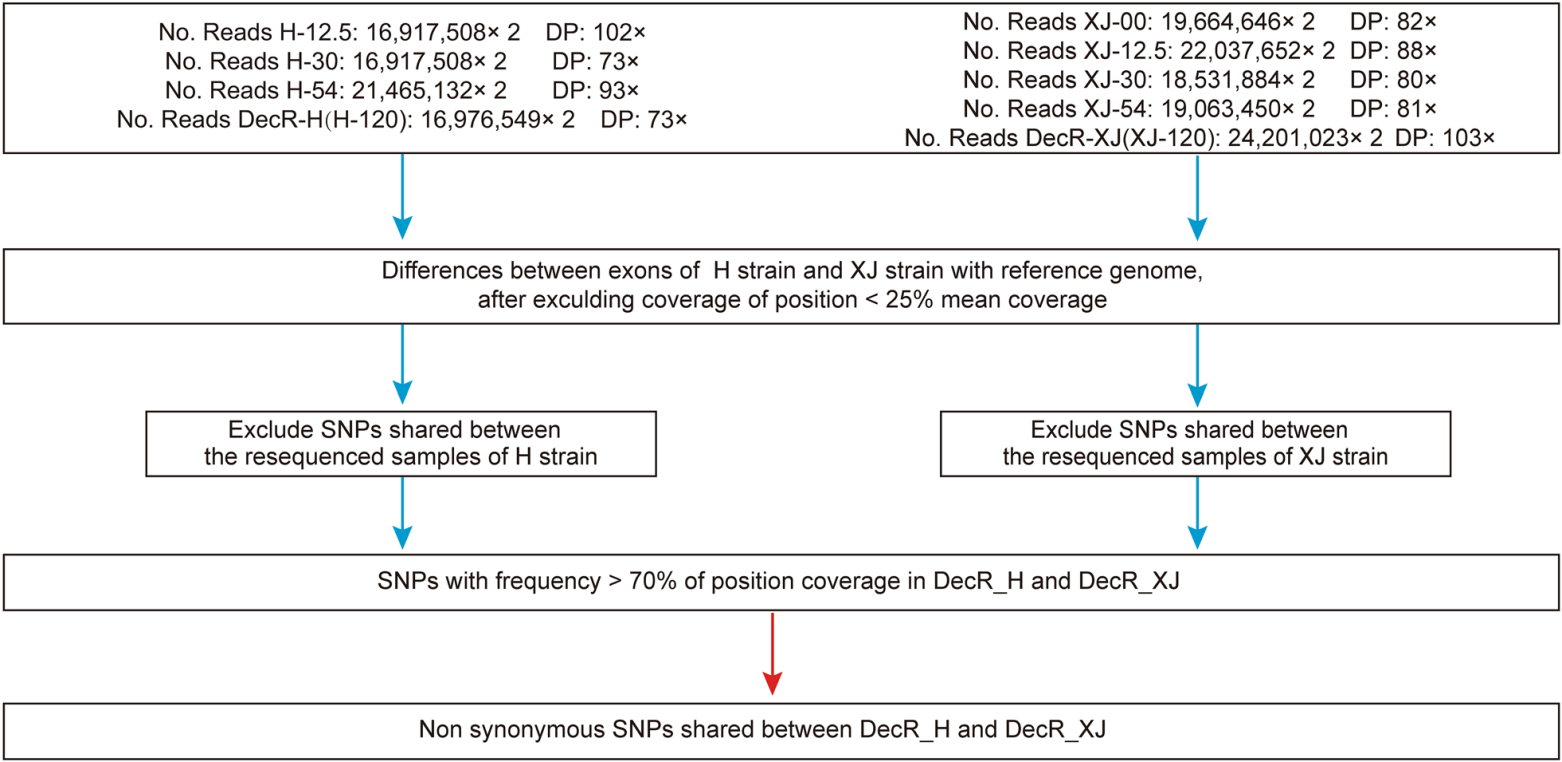
The statistics of the whole-genome sequence data and SNP-calling algorithm employed in this study. The statistics include the number of clean reads aligned to the reference genome and the bioinformatics workflow for non-synonymous SNP-calling. Concentration indicates the number of oocysts obtained at different decoquinate concentrations during the development of resistance. DP, sequencing depth; H, Houghton strain; SNP single nucleotide polymorphism; XJ, Xinjiang strain

The gene responsible for decoquinate resistance was analyzed using a specific method outlined in Fig. 2. In brief, SNPs located in the exon region were extracted from the high-quality reads using SnpSift [31] and filtered to remove those with low sequencing coverage by VCF Explorer [32]. SNPs intrinsic to parental strains were also excluded, and SNPs with sufficient allele frequency were screened. The extraction and annotation of genes containing missense mutations were performed by snpEff [31]. The complete annotated VCF file is provided as Additional file 1: File.

### Three-dimensional structure modeling and docking of the cytochrome* b*

The cytochrome *b* gene (ETH2_MIT00100) was analyzed by building three-dimensional (3D) structural models of different mutations using AlphaFold. The models were prepared using PyMOL Molecular Graphics System version 2.5.4 for educational purposes only (http://www.pymol.org). Additionally, the models were analyzed to predict ligand binding pockets using a neural network-based predictor (https://playmolecule.com/deepsite/). The structure of decoquinate was downloaded from the PubChem Database (https://pubchem.ncbi.nlm.nih.gov/compound/decoquinate, 29112). Ligand docking was performed with AutoDock Vina in PyMOL to gain a better understanding of the interactions between the ligand and the cytochrome* b* mutants.

## Results

### Three decoquinate-resistant *E. tenella* strains were obtained

Two strategies were employed to obtain drug-resistant *E. tenella* strains. The first strategy involved developing decoquinate resistance in sensitive parental strains (H and XJ strains; shown in Fig. 1A). Through 10 serial passages with progressively increasing drug levels, the chickens inoculated with oocysts of the passaged strains were able to shed large numbers of oocysts that were capable of sporulating when medicated with 120 mg/kg decoquinate (Additional file 2: Table S1; Additional file 3: Table S2). During the process, a plateau was encountered at the 30 mg/kg decoquinate concentration, characterized by sparse oocysts and a low rate of sporulation. However, once the plateau was crossed, even greater levels of drug seemed to have no effect, highlighting the qualitative nature of the resistance phenotype of decoquinate.

The second strategy involved isolating resistant strains from the field. The field isolates, with a record of decoquinate application failure for the prevention of coccidial infections, were propagated by inoculating chickens medicated with 120 mg/kg decoquinate. Subsequently, the *E. tenella* strain was isolated by inoculating a bird with a single oocyst of typical *E. tenella* morphology. As shown in Fig. 1B, only *E. tenella* was identified in the isolate by ITS-1 PCR methods. Thus, we obtained three *E. tenella* strains tentatively considered to be decoquinate resistant.

### The decoquinate resistance of three *E. tenella* strains were validated with animal trials

In order to further assess the decoquinate-resistant phenotype of the three strains, various criteria were used, including POAA based on weight gain, RLS based on intestinal lesion score and ROP based on oocyst production. As depicted in Fig. 1C–E, compared to the NNC groups, there was a significantly decreased weight gain in the INC and drugged groups, but no notable difference in weight gain between the INC and medicated groups (*df*=73, *P* < 0.05). Similarly, no significant reduction was found in medicated groups compared with INC groups in terms of intestinal lesion scores and cecal oocyst counts in the cecum (*P* < 0.05). As shown in Fig. 1F, further evaluation revealed that all three *E. tenella* stains were completely resistant to decoquinate when treated with 120 mg/kg (twice the recommended dose). Thus, we successfully acquired three decoquinate-resistant strains, which were collected from various regions and diverse models of developing resistance.

### Whole-genome sequencing reveals the correlation of mutations on cytochrome* b* to decoquinate resistance in three *E. tenella* strains

**Fig. 3:**
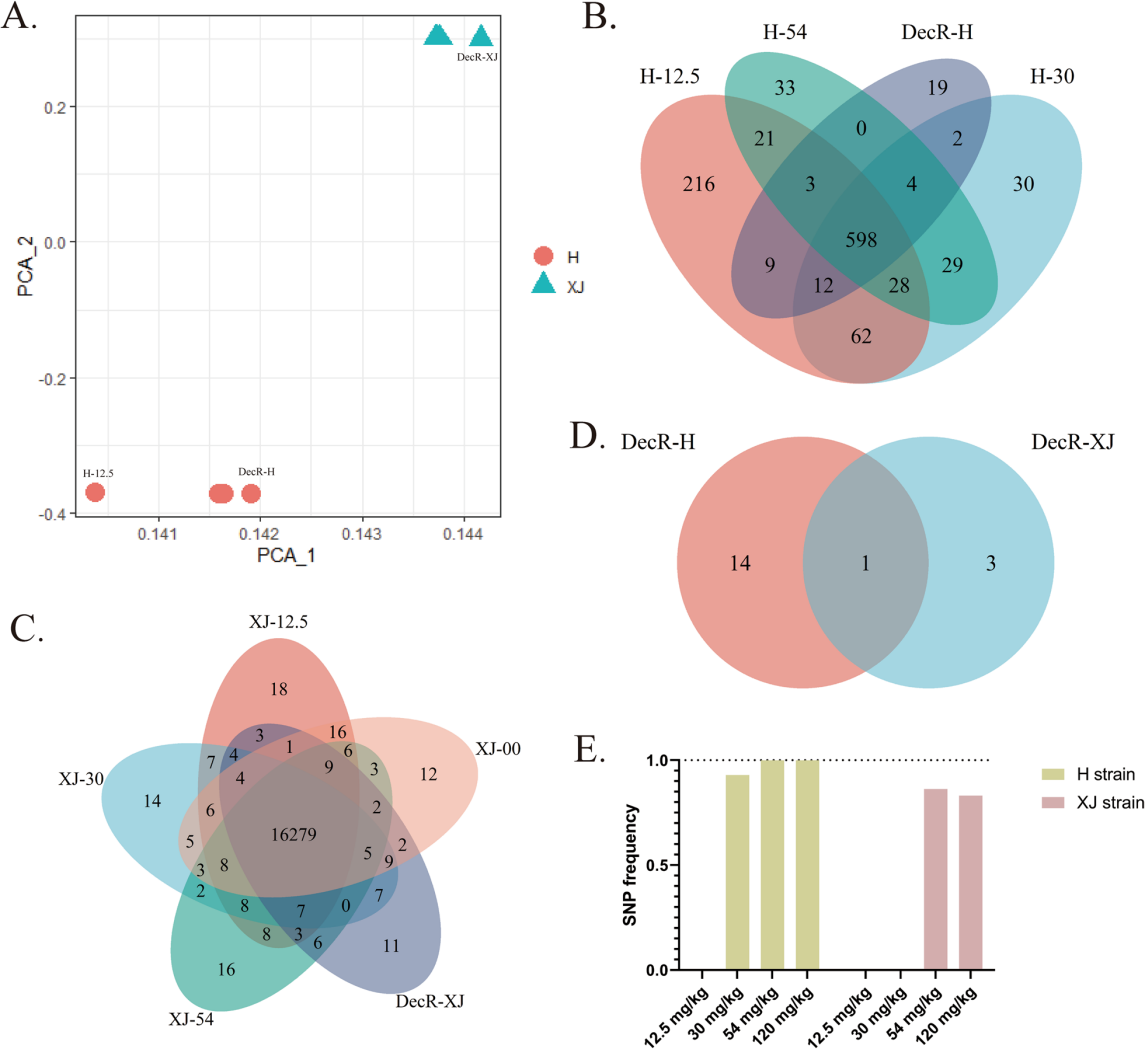
SNP analysis of decoquinate-resistant strains developed in the laboratory.** A** PCA analysis of SNPs in the sequencing samples of developing resistant strains. **B**,** C** Venn diagram of the SNPs in the H (**B**) and XJ (**C**) strains at increasing drug levels during the development of drug resistance.** D** Venn diagram of genes containing non-synonymous mutations shared between H-120 mg/kg (DecR_H) and XJ-120 mg/kg (DecR_XJ). **E** Occurrence and allele frequency of missense mutations in the cytochrome* b* gene during the development of drug resistance. DecR, Decoquinate resistance; H, Houghton strain; PCA, principal component analysis; SNP single nucleotide polymorphism; XJ, Xinjiang strain

To unravel the underlying key SNP(s) driving decoquinate resistance in *E. tenella*, whole-genome sequencing was performed on the drug-resistant parasites developed in the laboratory. The resulting clean reads were aligned to the reference genome, and the statistics of the alignment are depicted in Fig. 2. It is evident that each sequenced sample met the requirements for subsequent analysis. PCA based on genome-wide SNPs showed significant subpopulation segregation of the two resistant strains (Fig. 3A). However, the number of SNPs that emerged and became fixed during the development of resistance was much lower than the intrinsic SNPs in these strains (Fig. 3B, C). This issue led to some sequenced samples not being well distinguished from the others in the PCA analysis. Interestingly, the H strain had a lower number of intrinsic SNPs than the XJ strain, but a higher number of SNPs appeared during the development process in the H strain than in the XJ strain (Fig. 3B, C).

Previous studies have demonstrated that the gene encoding the drug target often acquires missense mutations in response to selection pressure, which is the primary molecular mechanism of drug resistance. Therefore, we focused on the non-synonymous mutations in the exon while excluding the intrinsic SNPs. The analysis of genes with missense mutations located in the protein coding region revealed that only ETH2_MIT00100 (cytochrome* b *[*cyt b*]) was shared between DecR_H and XJ strains (Fig. 3D). We then traced the frequency of mutant alleles of *cyt b* in resistant strains developed in the laboratory. The results demonstrated that the SNPs first appeared at 30 mg/kg decoquinate and became fixed during subsequent development (Fig. 3E), which is consistent with the pattern of oocyst reduction observed during the process of resistance development. These findings indicated a strong correlation between *cyt b* mutant alleles and resistance to decoquinate.

### Verification the correlation of *cyt b* mutation and decoquinate resistance

**Fig. 4:**
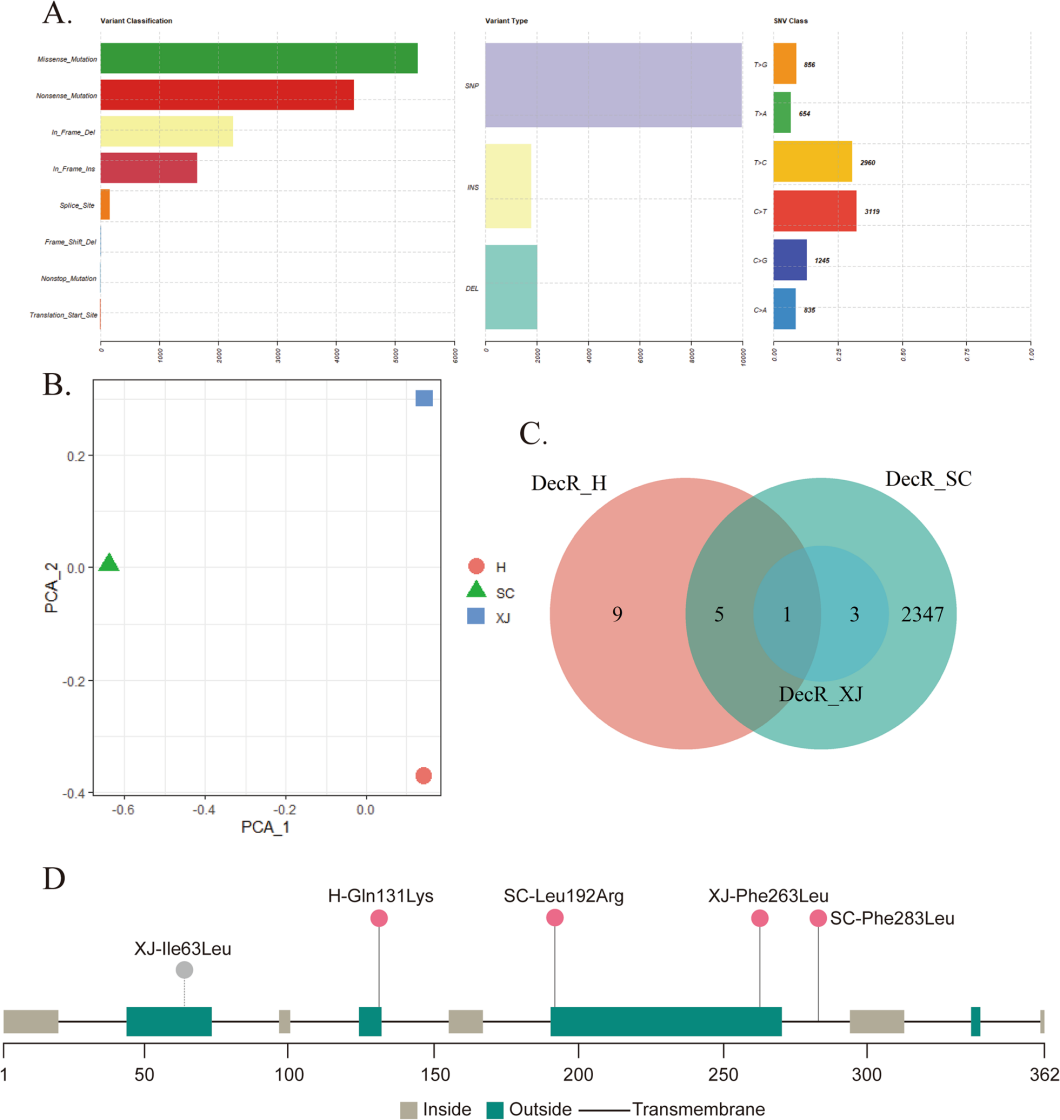
SNP analysis of DecR_SC and validation of *cyt b* mutations associated with decoquinate resistance.** A** A boxplot summary of next-generation sequencing data obtained from DecR_SC. **B** SNP-based PCA analysis of DecR_H, DecR_XJ and DecR_SC. **C** Venn diagram of genes containing non-synonymous mutations shared between DecR_H, DecR_XJ and DecR_SC. **D** Analysis of the transmembrane region of the *cyt b* gene and distribution of the non-synonymous mutations on *cyt b* in three drug-resistant strains. XJ-Ile63Leu is an intrinsic mutation in the XJ strain. *cyt b*, Cytochrome* b* gene; DecR, decoquinate resistance; H, Houghton strain; PCA, principal component analysis; SC, SC strain; SNP single nucleotide polymorphism; XJ, Xinjiang strain

To further verify the association between the mutation of *cyt b* and decoquinate resistance, we analyzed the next-generation sequencing data of the DecR_SC strain (Fig. 4A). The SNP-based PCA results demonstrated that the DecR_SC was distantly related to the laboratory-induced DecR_H and XJ strains, ensuring the reliability of the subsequent analysis (Fig. 4B). Missense mutations of *cyt b* were also observed in the DecR_SC strain, confirming our initial finding (Fig. 4C). However, different resistant strains presented unique SNPs in exon regions of the *cyt b* gene, suggesting that the SNPs arising in response to drug selection pressure may be linked to other factors, such as the intrinsic SNPs in *cyt b* (Fig. 4D; Additional file 4: Figure S1). The complex interplay between intrinsic and acquired mutations requires further investigation.

### Three-dimensional modeling and ligand docking shows decreased binding affinity of decoquinate onto mutated *cyt b*

**Fig. 5:**
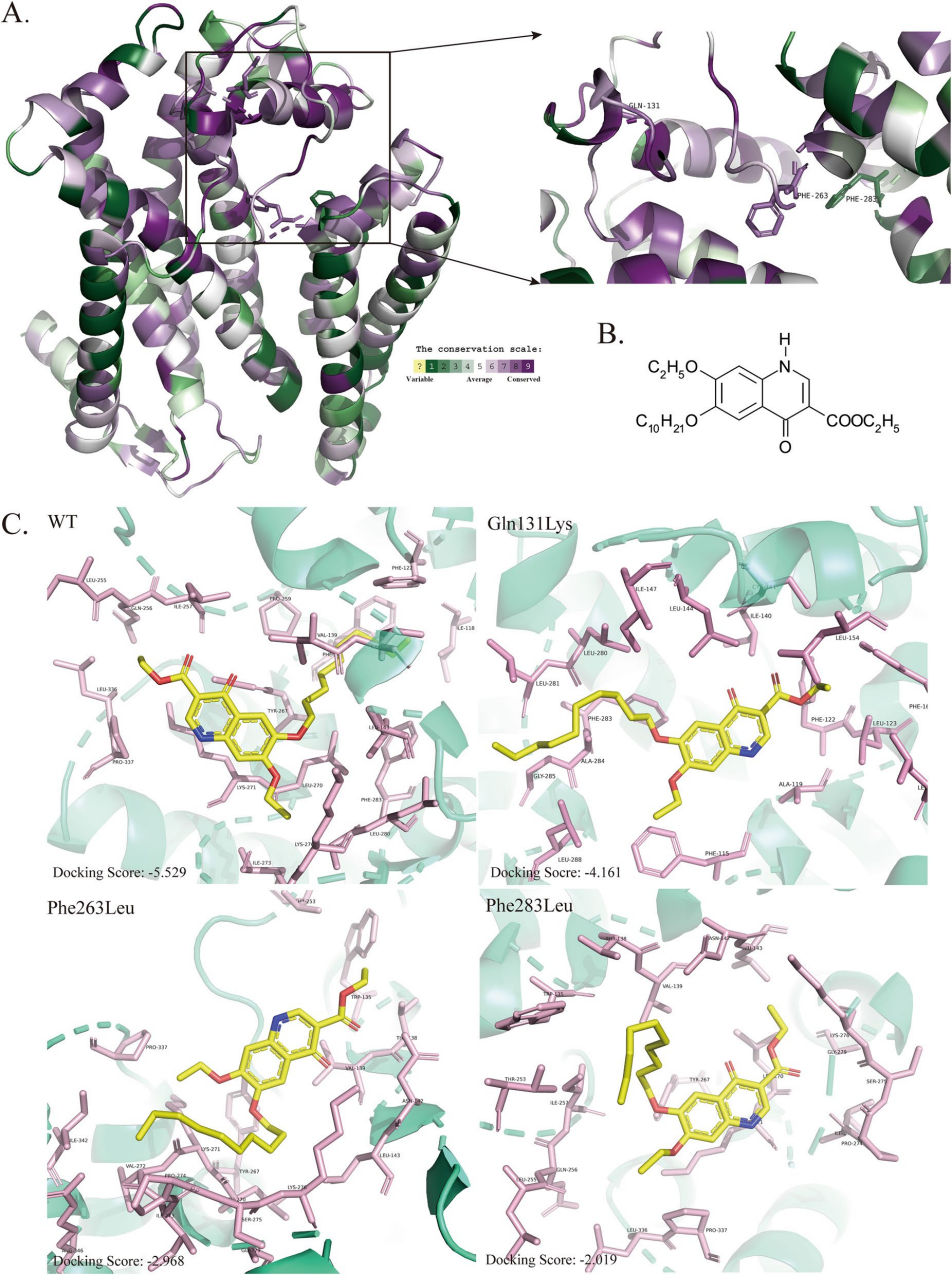
The model for *cyt b*-driven decoquinate resistance.** A**,** B** Conservation analysis and three-dimensional modeling of *cyt b* in reference sequences and presentation of mutation sites (**A**) and the chemical structures of decoquinate (**B**).** C** The docking results between decoquinate with wild-type and mutant *cyt b*, highlighting the amino acids shown in **A** in proximity of the ligand. The docking score is shown at the bottom left corner, with a higher score indicating reduced binding affinity of the protein with the ligand. *cyt b*, Cytochrome* b* gene

To gain a deeper understanding of the molecular mechanism behind the *cyt b* mutations that confer decoquinate resistance, we employed 3D modeling and molecular docking. Our analysis revealed that the cytochrome* b* protein consists of eight segments in the transmembrane region (Fig. 4D). Interestingly, 3D modeling results revealed that all missense mutations, except for Phe283Leu, were located on the outer mitochondrial inner membrane region. Further analysis showed that Gln131Lys, Phe263Leu and Phe283Leu were situated in the vicinity of the distinct ubiquinol oxidation site (*Q*_o_), causing slight alterations in the binding pockets through mutation (Additional file 5: File). Therefore, we speculate that Leu192Arg may not play a significant role in conferring decoquinate resistance. Moreover, the multiple sequence alignment of seven species of chicken *Eimeria*, *Plasmodium falciparum* and *T. gondii* (Fig. 5A; Additional file 6: Figure S2) indicated that the mutated loci were conserved, while the polymorphism at Phe283 was more likely attributed to *P. falciparum* and *Eimeria maxima*. Subsequently, we conducted ligand docking with the chemical structures of decoquinate (Fig. 5B) to the predicted binding pockets for mutant *cyt b*, as shown in Fig. 5C, which revealed changes in the binding pattern and a decrease in binding affinity. Hence, we conclude that mutations in cytochrome* b* play a crucial role in determining decoquinate resistance in *E. tenella* parasites.

## Discussion

In this study, we successfully acquired three decoquinate-resistant *E. tenella* strains with varying genetic backgrounds. Two of these strains (DecR_H and DecR_XJ) were developed through serial passages of the decoquinate-sensitive strains with progressively greater concentrations of drug, while the third strain (DecR_SC) was isolated from the field. After confirming the drug resistance through three indicators, we conducted whole-genome sequencing and found that mutations in the gene ETH2_MIT00100 (*cyt b*) were strongly associated with decoquinate resistance in *E. tenella*. Ligand docking experiments also confirmed that mutants had reduced molecular binding capacity.

The experimental development of resistance provides a robust approach for investigating coccidiostat resistance in *Eimeria*. Nearly all anticoccidial drugs, including decoquinate, have induced resistant strains in the laboratory. However, the ease of induction varies, with some drugs readily yielding resistance, while others prove more challenging [6]. Using laboratory-induced resistant strains can significantly narrow candidate genes. For example, a comparative analysis of one experimental artemisinin-resistant *P. falciparum* strain and its parental strain identified only seven genes with non-synonymous mutations linked to resistance [33]. In our study, the number of coding genes with non-synonymous mutations varied from 19 in DecR-H to four in DecR-XJ, with *cyt b* being the sole shared gene. Consequently, selecting strains with diverse genetic backgrounds for drug resistance development can further refine subsequent analyses, offering valuable insights into the genetics underpinning coccidial drug resistance.

Our findings are consistent with those of previous studies that reported associations between *cyt b* mutations and decoquinate resistance in other parasites, such as *P. falciparum* and *T. gondii* [34, 35]. However, it is worth noting that the in vitro development of decoquinate-resistant strains in *P. falciparum* and *T. gondii* in these studies was limited to asexual reproduction [34–36], while our study focused on determining the mutations responsible for decoquinate resistance throughout the entire life-cycle of *E. tenella* in chickens, including both asexual and sexual reproduction. Despite this difference, our results consistently showed that *cyt b* was the only gene with missense mutations in all three drug-resistant strains, highlighting its central role in decoquinate resistance. Non-synonymous mutations in a gene conferring parasite resistance via reduced binding affinity have been demonstrated in *Plasmodium* studies [37]. However, *cyt b* is a mitochondria-encoded gene and, consequently, its genetics and mutation thresholds are complex. We attempted to determine the heterogeneity threshold of *cyt b* mutations conferring drug resistance, but found that the proportion of mutants, once present, reached very high value. This may be the result of drug selection pressure and intrapopulation competition.

In the present study, we identified three novel mutations, namely Gln131Lys, Phe263Leu and Phe283Leu, in the extracellular binding pocket of the *cyt b* gene of *E. tenella* decoquinate-resistant strains. These specific amino acid substitutions were located within the Q_o_ pocket region of *cyt b*, which represents the decoquinate binding site. Notably, while these mutations were present in decoquinate-resistant *E. tenella* strains, our analysis did not detect the same non-synonymous mutations in these strains.

One potential explanation for the observed polymorphic mutations could be intrinsic differences between the strains. Notably, while the H strain served as the reference strain that was not exposed to any drugs [38], the XJ and SC strains were isolated from broiler farms, which could have contributed to the intrinsic genetic variations in these strains. Although we have determined the sensitivity or resistance of the strains to decoquinate, it remained still uncertain whether the two field strains were resistant to other drugs and whether the intrinsic mutations in *cyt b* have an impact on the arising SNPs in response to decoquinate pressure.

Another possible explanation for the polymorphic mutations is the presence of fitness costs associated with drug resistance, as observed in *P. falciparum* [39]. Mutations in *cyt b* that confer atovaquone resistance have been reported to impose a substantial fitness cost during sexual stage development in *Anopheles* mosquitoes, suggesting that resistance is not easily transmissible in the field [40]. Although there was little information about fitness costs of coccidia resistance was provided in this study, decoquinate-resistant strains were found to be at a disadvantage when equal numbers of sensitive and resistant oocysts were introduced into a floor pen of chickens [18]. Therefore, we speculate that decoquinate-resistant strains harboring different mutations may have a better ability to complete the life-cycle, potentially reducing the fitness cost associated with *cyt b* mutations.

Atovaquone, an anti-malarial agent used in combination with proguanil, acts as a competitive inhibitor of the quinol oxidation site of the mitochondrial cytochrome *bc*_*1*_ (*cyt bc*_*1*_) complex [41]. Docking studies have suggested that decoquinate belongs to the quinol oxidation inhibitor class [34]. Previous studies on *Plasmodium berghei* have reported various mutations in the *cyt b* gene, such as Met133Ile, Leu144Ser, Leu271Val, Lys272Arg, Tyr268Cys, Tyr268Ser, Tyr286Asn and Val284Phe, using a mouse model with continuous atovaquone pressure. Of these, Tyr268Ser was the most prevalent variant of five mutations in *P. falciparum* [42, 43]. These results suggest that it is possible that the mutations in *cyt b* in response to the inhibitor are polymorphic.

The hydrophobic subunit of cytochrome *b* is an essential component of the *cyt bc*_*1*_ complex, which functions as a proton-motive ubiquinol [44] and plays a crucial role in oxidizing ubiquinol (QH_2_) to ubiquinone (Q) and donating electrons to cytochrome* c* oxidase. In parasitic protozoa, including *E. tenella*, dihydroorotate dehydrogenase (DHODH) also acts as an essential enzyme for the de novo synthesis of pyrimidines, which is the exclusive pathway to synthesize pyrimidines [45, 46]. However, in one study, the addition of uracil to the medium did not have a substantial effect on the in vitro anti-parasite activity of decoquinate, suggesting that de novo pyrimidine synthesis was not the primary biochemical target of decoquinate in *T. gondii* [36]. On the other hand, the transgenic *P. falciparum* parasites expressing the type 1A DHODH from *Saccharomyces cerevisiae* (*Sc*DHOHD), which utilizes fumarate as the final electron acceptor in the pyrimidine biosynthesis pathway and is independent of the *cyt bc*_*1*_ complex, showed a significant resistance to decoquinate and atovaquone against the transgenic line relative to the parental line [34]. It has been reported that mitochondrion contribution to the whole ATP synthesis in blood stages remains minimal [47]. Hence, decoquinate blocks electron transport from QH_2_ to *cyt bc*_*1*_, inhibiting the synthesis of pyrimidines. As for *E. tenella*, it is still unclear whether pyrimidine biosynthesis is blocked by the interference of *cyt b* with decoquinate.

In order to more definitively verify that mutations in the *cyt b* gene determine the development of decoquinate resistance, implementing gene editing represents a more direct approach. However, *cyt b* is an extra-nuclear gene encoded by mitochondria, and its editing using the CRISPR/Cas9 system remains quite challenging. The difficulty in editing mitochondrial DNA (mtDNA) using CRISPR/Cas9 stems from efficiently delivering the Cas9 protein and guiding RNA into the mitochondria. Mitochondria possess unique structures and membranes, which impede the CRISPR/Cas9 components from entering and targeting the mtDNA. The ability of genomic RNA (gRNA) to enter mitochondria remains controversial [48]. Of course, the current mitochondria-based TALE-DddA system may provide a more reliable methodology [49]. Realizing the editing of the mitochondrial genome of coccolithophores will likely prove an extremely challenging endeavor.

Thus, in order to develop a more comprehensive and precise detection platform for decoquinate-resistant strains, it is imperative that future research focus on analyzing a larger sample size of field-resistant strains. Such an approach would help to ensure the accuracy and effectiveness of the detection method.

## Conclusions

The aim of the present study was to identify the genetic mutations associated with decoquinate resistance in *E. tenella* parasites. Our findings reveal a strong correlation between *cyt b* mutant alleles and decoquinate resistance, highlighting the crucial role of *cyt b* mutations in conferring resistance to decoquinate. Hence, *cyt b* mutations could serve as a valuable molecular marker for detecting the presence of decoquinate resistance.

### Supplementary Information

**Additional file 1.** The complete SNP file, in VCF format, was utilized in this study. The file generated from the NGS data was obtained after calling and filtering SNPs.

**Additional file 2: Table S1.** Development of decoquinate resistance in the *E. tenella* Houghton strain.

**Additional file 3: Table S2.** Development of decoquinate resistance in the *E. tenella* Xinjiang strain.

**Additional file 4: Figure S1.** The multiple sequence alignment of yhr *cyt b *gene sequences between the parental strains, induced decoquinate-resistant strains of H and XJ strains, and the sequence of *cyt b* from the SC strain.

**Additional file 5.** The prediction of various mutant cytochrome* b *ligand binding pockets was conducted using DeepSite.

**Additional file 6: Figure S2.** The multiple sequence alignment of amino acids for* Toxoplasma gondii*, *Plasmodium falciparum *and seven chicken *Eimeria* species. The detected mutations are presented at the top of the sequence, and the conserved scale is shown in the lower-left corner.

## Data Availability

The article’s supporting datasets, which validate the conclusions, have been included as Additional file 1.

## Abbreviations

CTAB: Cetyltrimethylammonium bromide
GSR: Growth and survival ratio
mtDNA: Mitochondrial DNA
PBS: Phosphate buffer saline
POAA: Percentage of optimum anticoccidial activity
RLS: Reduction of lesion scores
ROP: Relative oocyst production (ROP)
SNP: Single nucleotide polymorphism
