# Supplementary figures and images for: Distinct non-synonymous mutations in cytochrome b highly correlate with decoquinate resistance in apicomplexan parasite Eimeria tenella
Source: Parasit Vectors. 2023 Oct 17;16:365. doi: 10.1186/s13071-023-05988-7 (PMC10583425; doi:10.1186/s13071-023-05988-7)

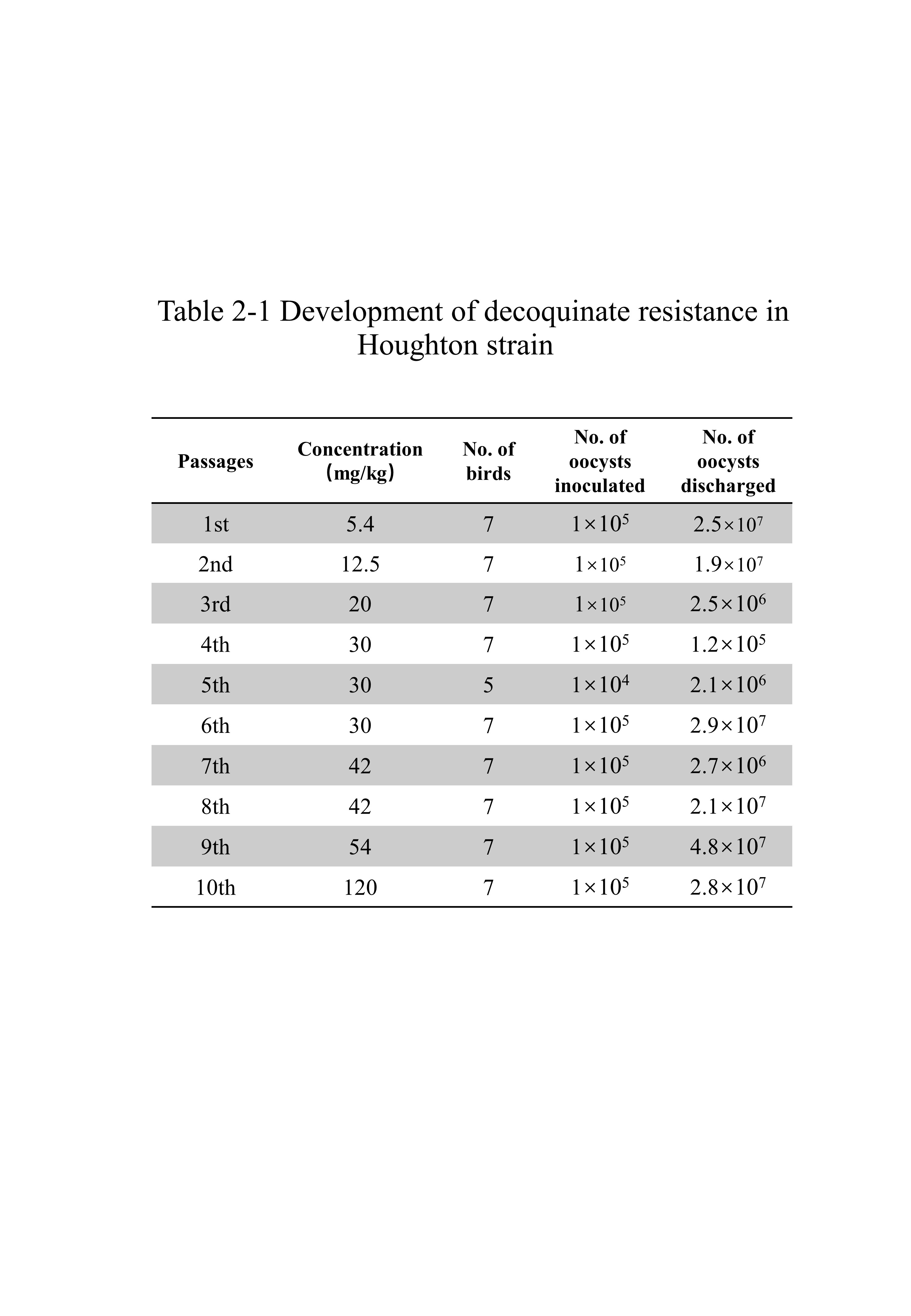

Supplement: Supplementary file 2 — Additional file 2: Table S1. Development of decoquinate resistance in the E. tenella Houghton strain. [file 13071_2023_5988_MOESM2_ESM.jpg]

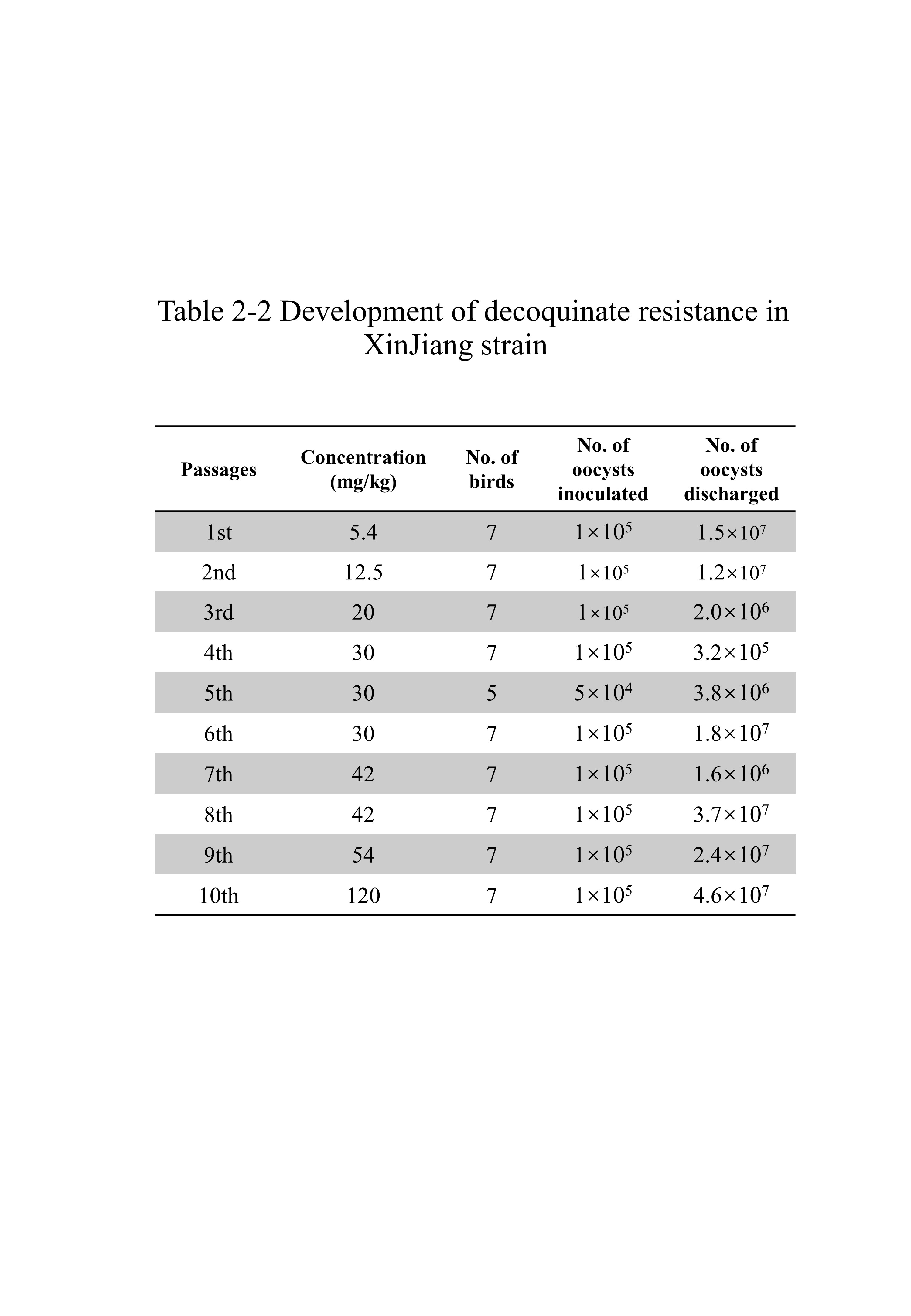

Supplement: Supplementary file 3 — Additional file 3: Table S2. Development of decoquinate resistance in the E. tenella Xinjiang strain. [file 13071_2023_5988_MOESM3_ESM.jpg]

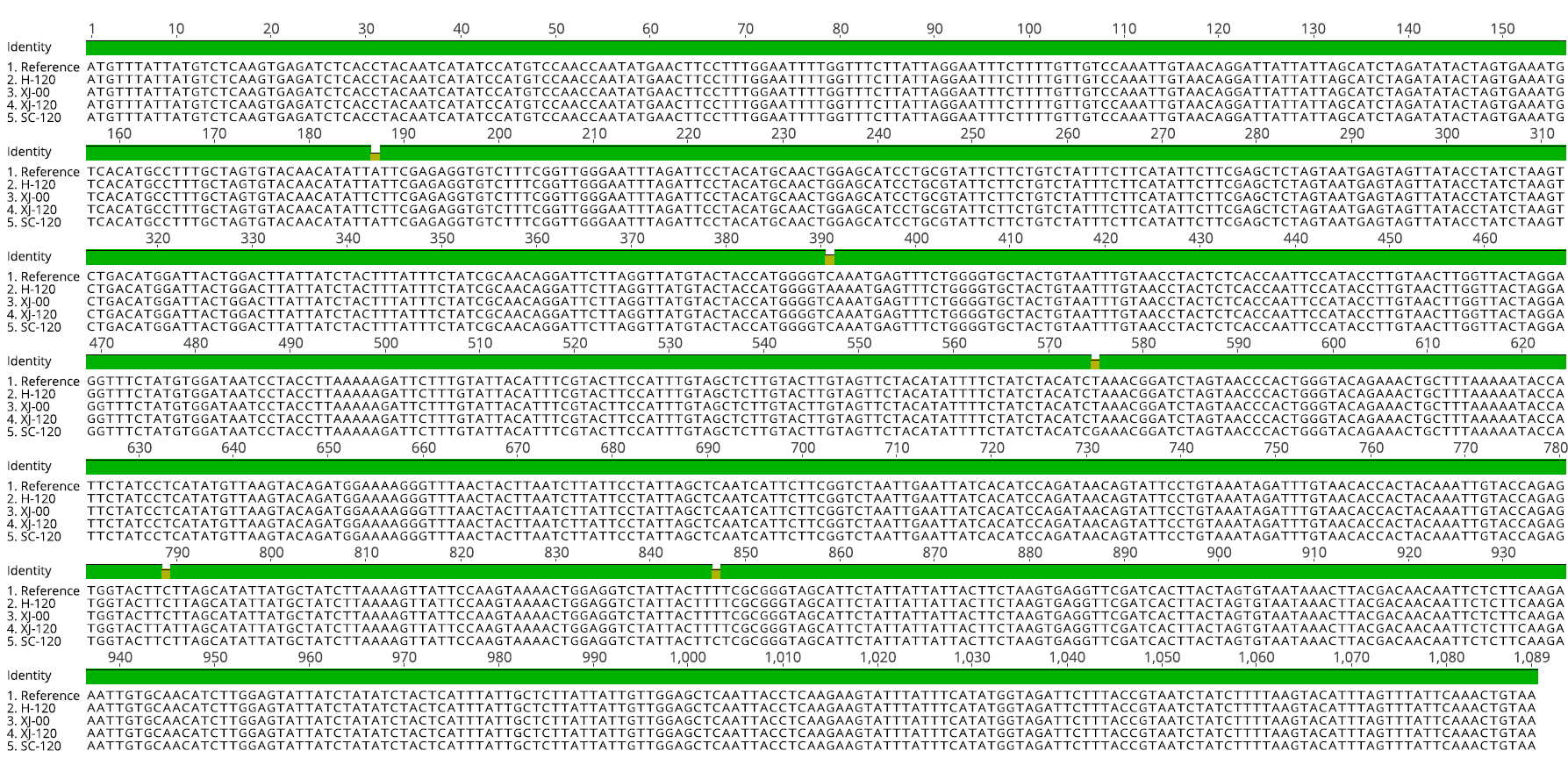

Supplement: Supplementary file 4 — Additional file 4: Figure S1. The multiple sequence alignment of yhr cyt b gene sequences between the parental strains, induced decoquinate-resistant strains of H and XJ strains, and the sequence of cyt b from the SC strain. [file 13071_2023_5988_MOESM4_ESM.png]

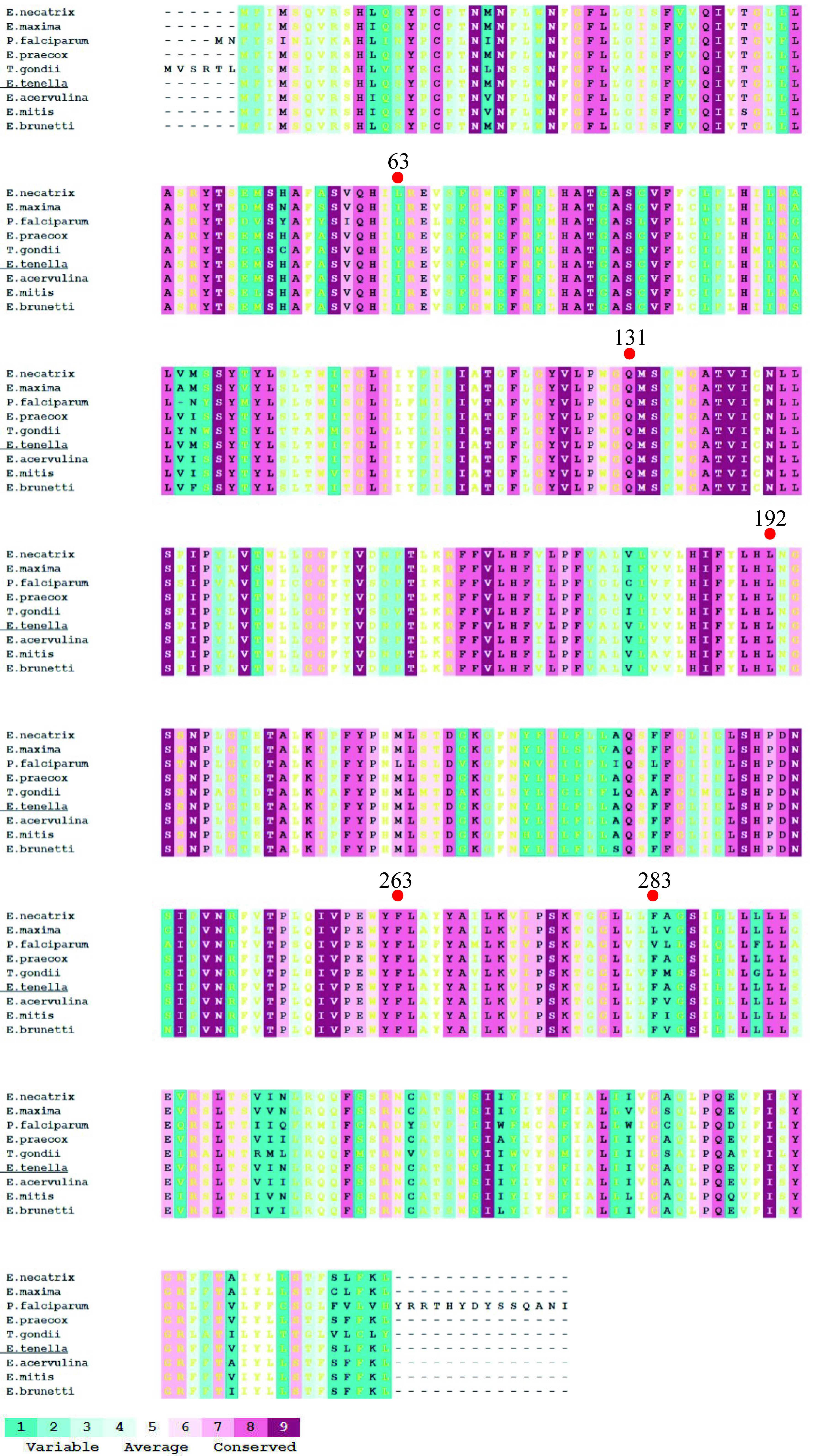

Supplement: Supplementary file 6 — Additional file 6: Figure S2. The multiple sequence alignment of amino acids for Toxoplasma gondii, Plasmodium falciparum and seven chicken Eimeria species. The detected mutations are presented at the top of the sequence, and the conserved scale is shown in the lower-left corner. [file 13071_2023_5988_MOESM6_ESM.tif]
